# Antimicrobial Resistance Across the Urban Wastewater Continuum: A One Health Assessment Using High-Throughput qPCR

**DOI:** 10.3390/antibiotics15070669

**Published:** 2026-07-08

**Authors:** Douha Shouqair, Rashed Alghafri, Subham Verma, Mohammed Naji, Abdulla Albastaki, Fatima Al Dhaheri, Mahmood Y. Hachim, Rania Nassar, Ahmed A. Shibl, Jorge Rodríguez, Dean Everett, Richard Goering, Mushtaq Khan, Abiola Senok

**Affiliations:** 1 College of Medicine, Mohammed Bin Rashid University of Medicine and Health Sciences, Dubai P.O. Box 505055, United Arab Emirates; douha.shouqair@students.mbru.ac.ae (D.S.); subham.verma@dubaihealth.ae (S.V.); mahmood.almashhadani@dubaihealth.ae (M.Y.H.); rania.nassar@dubaihealth.ae (R.N.); 2 General Department of Forensic Science and Criminology, Dubai Police, Dubai P.O. Box 1493, United Arab Emirates; r.alghafri@dubaipolice.gov.ae (R.A.); m.naji4566@gmail.com (M.N.); albastaki94@gmail.com (A.A.); 3 Center for Microbial Sciences, Mohammed Bin Rashid University of Medicine and Health Sciences, Building 14, Dubai Healthcare City, Dubai P.O. Box 505055, United Arab Emirates; 4 Department of Pediatrics, College of Medicine and Health Sciences, United Arab Emirates University, Al-Ain P.O. Box 15551, United Arab Emirates; fatimaald@uaeu.ac.ae; 5 Public Health Research Center, New York University Abu Dhabi, Abu Dhabi P.O. Box 129188, United Arab Emirates; ahmed.shibl@nyu.edu; 6 Department of Chemical and P. Engineering, Khalifa University, Abu Dhabi P.O. Box 127788, United Arab Emirates; jorge.rodriguez@ku.ac.ae; 7 Department of Public Health and Epidemiology, College of Medicine and Health Sciences, Khalifa University, Abu Dhabi P.O. Box 127788, United Arab Emirates; dean.everett@ku.ac.ae; 8 Biotechnology Center, Khalifa University, Abu Dhabi P.O. Box 127788, United Arab Emirates; 9 Infection Research Unit, Khalifa University, Abu Dhabi P.O. Box 127788, United Arab Emirates; 10 Department of Medical Microbiology and Immunology, Creighton University School of Medicine, Omaha, NE 68178, USA; richardgoering@creighton.edu; 11 Department of Medical Microbiology and Immunology, College of Medicine and Health Sciences, United Arab Emirates University, Al-Ain P.O. Box 15551, United Arab Emirates; mushtaq.khan@uaeu.ac.ae; 12 Zayed Bin Sultan Center for Health Sciences, United Arab Emirates University, Al-Ain P.O. Box 17666, United Arab Emirates; 13 School of Dentistry, Cardiff University, Cardiff CF14 4XY, UK

**Keywords:** wastewater-based surveillance, antimicrobial resistance, high throughput-qPCR, wastewater treatment plant, One Health

## Abstract

**Background**: Wastewater systems provide an integrated One Health perspective on antimicrobial resistance but remain uneven globally, with limited data from rapidly urbanizing and highly connected regions such as the Arabian Gulf. **Methods**: An eight-month prospective study was conducted in Dubai, United Arab Emirates, with monthly sampling from nine community and two hospital nodes and two wastewater treatment plants (WWTP). Samples were analysed using high-throughput quantitative PCR (HT-qPCR; Resistomap, Finland) with a 72-target One Health gene panel. **Results**: Across the 120 samples analyzed, the number of detected gene targets ranged from 26 to 68 genes, with the highest diversity in hospital wastewater and the lowest in WWTP effluent. Pathogen-associated markers were detected in all sources, with enterococci, *Escherichia coli*, and *Klebsiella pneumoniae* predominant. Hospital wastewater showed broader pathogen-associated gene markers, including those linked to *Acinetobacter baumannii* and *Pseudomonas aeruginosa*. Antibiotic resistance genes (ARGs) associated with macrolide–lincosamide–streptogramin B, tetracycline, and aminoglycoside resistance were widespread. Community and influent samples were dominated by *msrE*, *tet(M)*, and aminoglycoside resistance genes, whereas hospital wastewater showed the highest ARG burden, including enrichment of *aac(6′)-Ib*, *qnrS2*, *bla*_GES_, *bla*_TEM_, *bla*_KPC-2_, and *bla*_IMP-1_. Several ARGs, including *mcr-1*, persisted in WWTP effluent. Mobile genetic elements (MGEs) were ubiquitous, with integron-associated markers prominent in WWTP effluent. ARG–MGE network analysis demonstrated extensive co-occurrence, with MGEs as central hubs linking multiple ARGs. **Conclusions**: Wastewater captures distinct resistome profiles across urban compartments, supporting its role for AMR surveillance. The persistence of ARGs and MGEs in WWTP effluent highlights the potential for environmental dissemination, through reuse of treated wastewater.

## 1. Introduction

Antimicrobial resistance is one of the most pressing global public health challenges [1]. Recent estimates indicate that AMR was associated with approximately 4.95 million deaths globally in 2019, including 1.27 million deaths directly attributable to resistant infections [2]. A more recent systematic analysis of the global burden of bacterial AMR from 1990 to 2021 estimated 1.14 million attributable deaths and 4.71 million associated deaths in 2021, with annual attributable mortality projected to increase to approximately 1.91 million by 2050 [3]. Antibiotic resistance genes (ARGs) are naturally occurring in environmental bacteria, having evolved over billions of years [4]; however, widespread use and misuse of antibiotics in human healthcare, agriculture, and livestock production have significantly accelerated their selection, enrichment, and dissemination across microbial communities [5].

While clinical surveillance remains essential for monitoring infections caused by resistant pathogens [6], it captures only a small fraction of the circulating resistome because it primarily reflects cases presenting to healthcare settings. Resistance genes circulating within commensal microbial communities of asymptomatic individuals, which may persist and disseminate through horizontal gene transfer, remain underestimated. In recent years, increasing attention has shifted toward wastewater-based surveillance approaches [6] within the One Health framework to capture microbial inputs across diverse niches [7]. Wastewater-based surveillance provides a population-level perspective and has been shown to serve as an early warning system [8]. This is particularly pertinent as mobile genetic elements (MGEs) such as plasmids, integrons, and transposons in wastewater can facilitate horizontal gene transfer within microbial communities [9]. Residual antimicrobial compounds originating from domestic, hospital, and industrial sources may exert selective pressure on microbial populations, potentially promoting the persistence and spread of resistance genes [10]. In addition, biofilm formation along pipe surfaces provides stable microbial habitats that can facilitate ARG accumulation and horizontal gene transfer within sewer networks [11]. While community-derived wastewater provides a population-level snapshot of AMR circulation, integrating contributions from both symptomatic and asymptomatic carriers [12], hospital wastewater represents a distinct and often enriched reservoir of clinically relevant resistance genes due to high antibiotic use and concentrated patient populations [13].

Approaches for investigating AMR in wastewater include culture-based methods, targeted molecular assays, and shotgun metagenomics [14]. Culture-based approaches enable phenotypic characterization of resistant bacteria but are limited to cultivable organisms and therefore underestimate microbial and resistome diversity in complex environmental samples [15]. Shotgun metagenomics provides comprehensive, untargeted insight into wastewater resistomes by enabling detection of both known and potentially novel ARGs, characterization of microbial community composition, and assessment of the genomic context of resistance determinants, including their potential association with MGEs but remains resource-intensive and computationally demanding for large-scale surveillance [16]. High-throughput quantitative PCR (HT-qPCR) has emerged as a widely used alternative, enabling detection and quantification of large ARG panels across numerous samples [17,18,19]. HT-qPCR offers rapid processing, lower cost, and scalability, making it particularly suitable for longitudinal monitoring and large-scale surveillance.

Despite increasing interest in wastewater-based AMR surveillance, its application in the Arabian Gulf region remains limited. To address this gap, the present study applied a One Health framework to evaluate the distribution and interaction of resistance determinants across interconnected urban wastewater compartments. A multi-source longitudinal sampling design combined with HT-qPCR was used to characterise AMR patterns across urban wastewater systems. Specifically, the study aimed to evaluate the distribution of pathogen markers, ARGs, and MGEs across wastewater sources (community, hospital, and WWTP), assess spatial and temporal resistome variability, and evaluate the utility of HT-qPCR as a scalable approach for wastewater-based AMR surveillance in an urban setting.

## 2. Results

A total of 120 wastewater samples were collected over the study period. Across all wastewater samples, 26–68 gene targets were detected. Hospital wastewater exhibited the highest number of detectable targets (62–68), followed by WWTP influent (60–67) and community wastewater (45–67). WWTP effluent samples showed the lowest detection range (26–63). Complete gene-level quantification is provided in Supplementary Data S2.

### 2.1. Pathogen-Associated Marker

**Figure 1:**
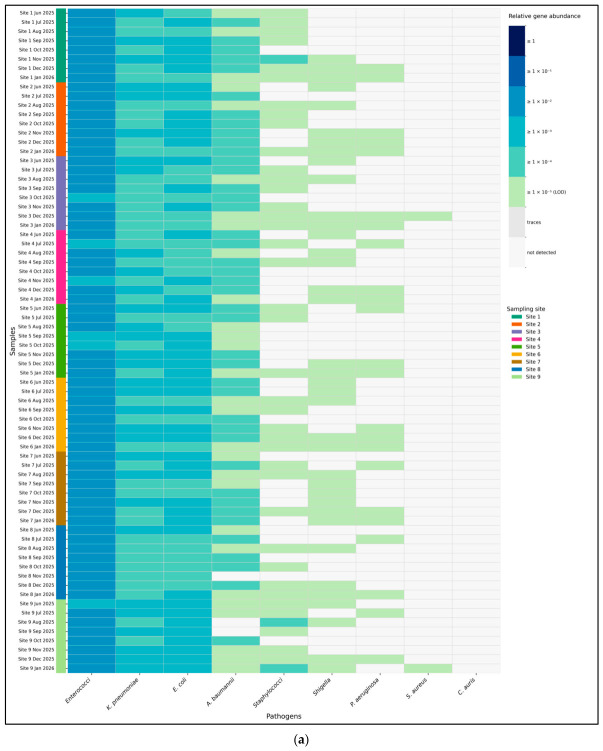

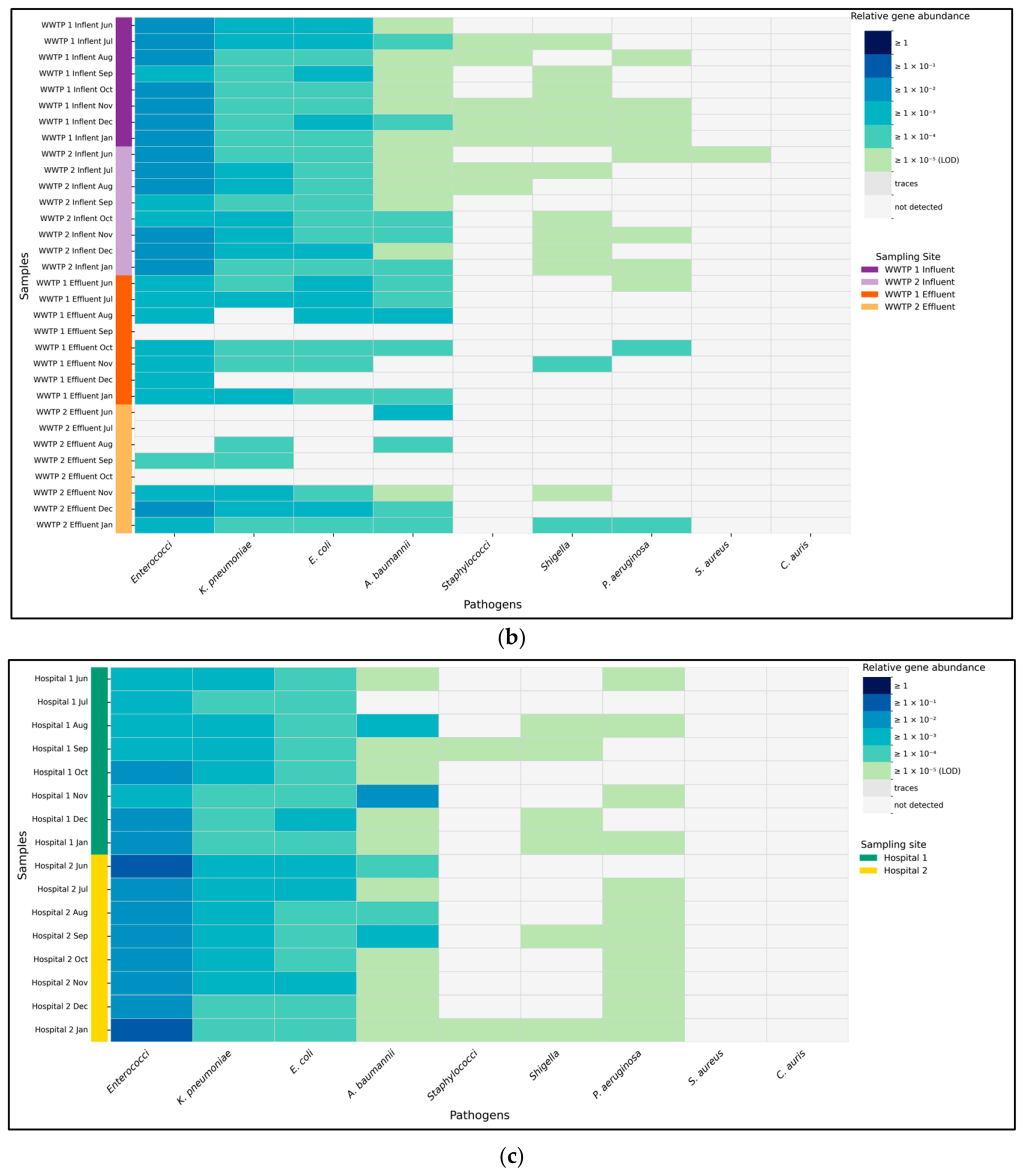
Distribution of relative abundance of pathogen-associated marker genes across wastewater sources. Heatmap showing the relative abundances of pathogen marker genes based on HT-qPCR profiling, across wastewater sources: (**a**) community wastewater, (**b**) WWTP influent and effluent; and (**c**) hospital wastewater. Rows represent individual samples and columns represent pathogen-associated marker genes. Relative gene abundance is indicated by a colour gradient from low (light green) to high (dark blue). Samples are grouped by sampling source, as indicated by the color bars.

Pathogen-associated markers were detected across all wastewater sources, with clear differences in prevalence and relative gene abundance (Figure 1). In community wastewater, enterococci, *Klebsiella pneumoniae*, and *Escherichia coli* were detected in all samples and showed the highest relative gene abundance, with enterococci predominating (mean ± SD: 3.06 × 10^−2^ ± 1.67 × 10^−2^). Detection frequencies were lower for markers targeting *A. baumannii* (*n*/*N* = 69/72), *Staphylococcus* spp. (*n*/*N* = 41/72), *Shigella* (*n*/*N* = 37/72), *P. aeruginosa* (*n*/*N* = 23/72), and *Staphylococcus aureus* (*n*/*N* = 2/72) (Figure 1a). In WWTP influent, the three pathogens detected at high relative gene abundance were enterococci (mean ± SD: 1.89 × 10^−2^ ± 7.85 × 10^−3^), *K. pneumoniae* (mean ± SD: 1.58 × 10^−3^ ± 2.10 × 10^−3^) and *E. coli* (mean ± SD: 1.01 × 10^−3^ ± 3.93 × 10^−4^). Although *A. baumannii* was detected in all influent samples, its relative abundance remained low (mean ± SD: 1.06 × 10^−4^ ± 1.11 × 10^−4^). *S. aureus* was detected in only one influent sample. WWTP effluent samples showed an overall reduction in the detection frequency of pathogen-associated markers. enterococci and *E. coli* were universally detected in all WWTP effluent samples, whereas other pathogens were detected sporadically or were absent. Although detection frequency declined in WWTP effluent, the relative abundance of the markers detected ranged from mean ± SD: 4.62 × 10^−3^ ± 4.33 × 10^−3^ to 3.05 × 10^−5^ ± 7.42 × 10^−5^ which was within the same order of magnitude as those observed in influent samples (Figure 1b). In hospital wastewater, enterococci, *K. pneumoniae*, and *E. coli* exhibited prevalence and relative gene abundance patterns similar to those observed in community wastewater and WWTP influent (Figure 1). Majority of hospital samples had detection of markers for *A. baumannii* (*n*/*N* = 15/16) and *P. aeruginosa* (*n*/*N* = 11/16). The relative gene abundances for *A. baumannii* (mean ± SD: 1.15 × 10^−3^ ± 3.32 × 10^−3^) and *P. aeruginosa* (mean ± SD: 2.34 × 10^−5^ ± 2.10 × 10^−5^) in hospital wastewater was comparable to those observed in other wastewater sources. *S. aureus* was not detected in hospital wastewater.

### 2.2. Antimicrobial Resistance Genes (ARGs) and Mobile Genetic Elements (MGEs)

**Figure 2:**
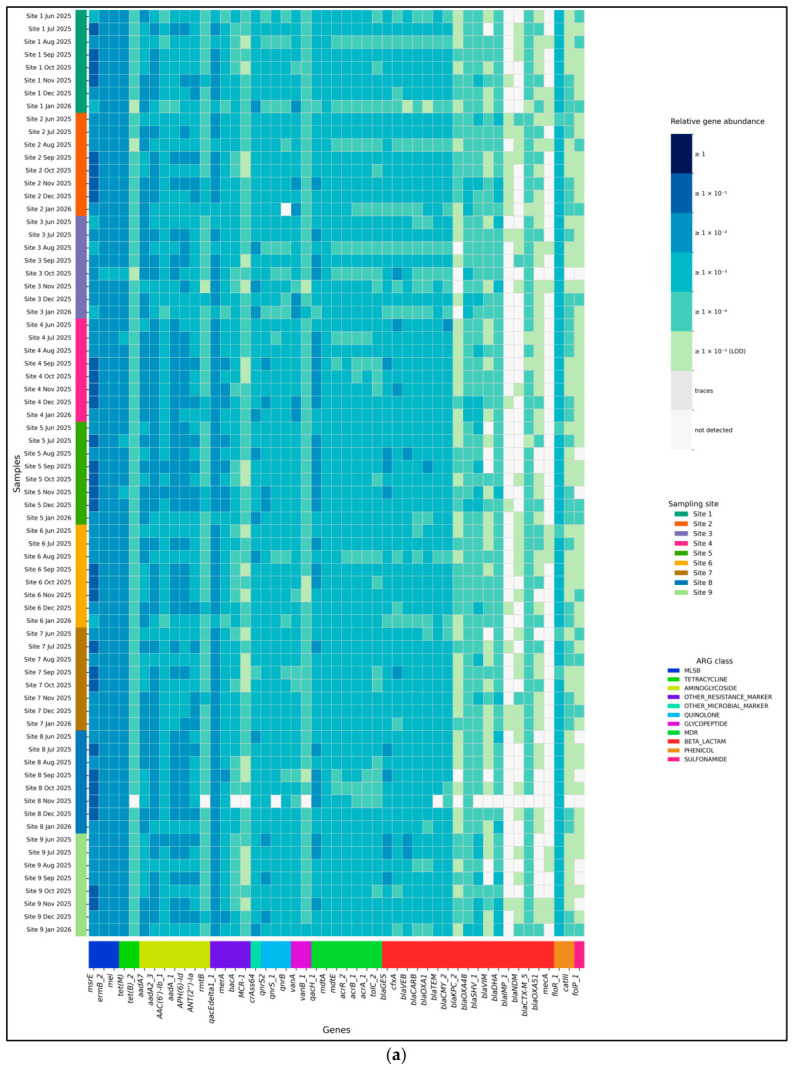

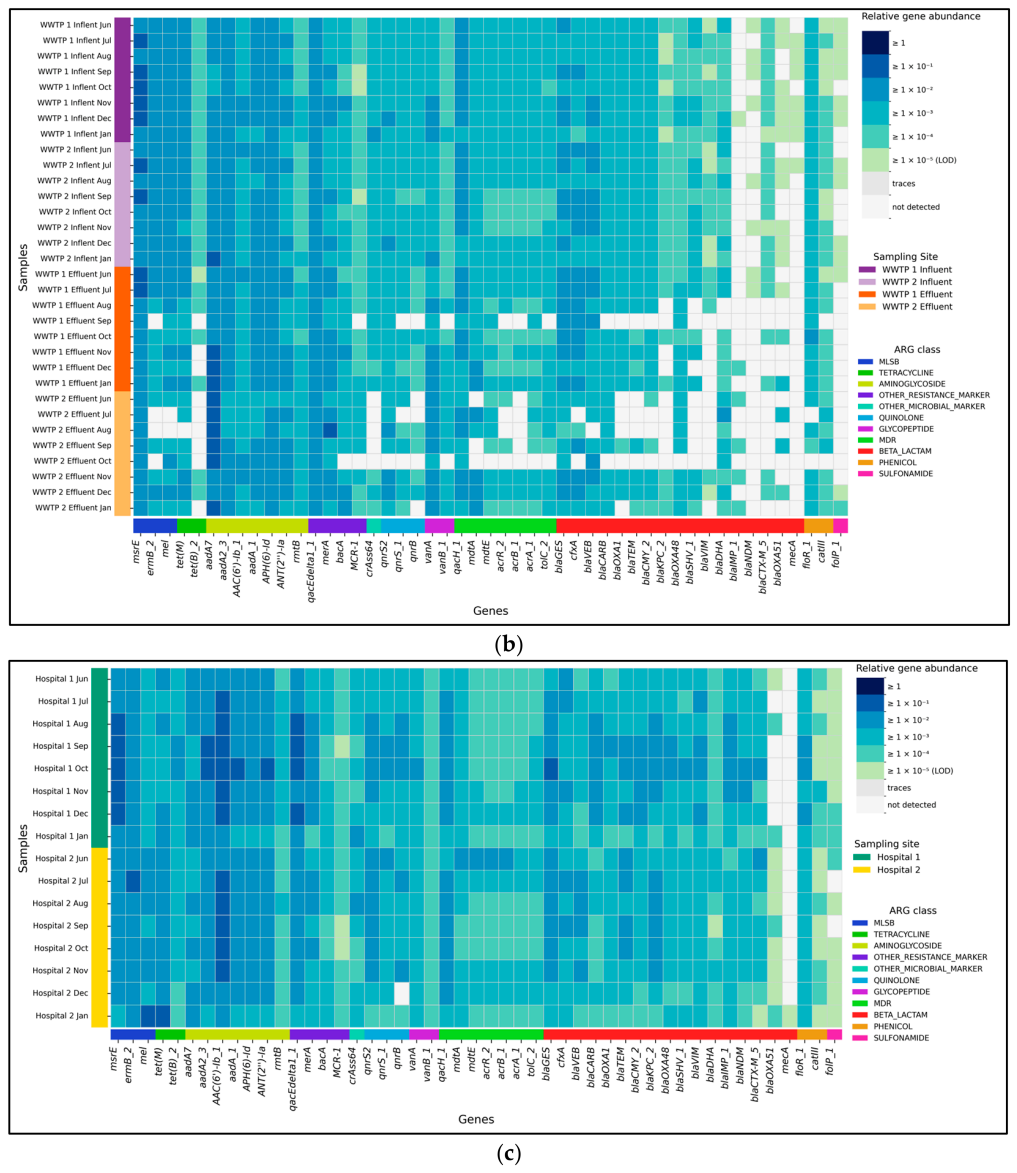
Distribution of relative abundance of antibiotic resistance genes across wastewater sources. Heatmap showing the relative abundances of antibiotic resistance genes (ARGs) based on HT-qPCR profiling, across wastewater sources: (**a**) community wastewater, (**b**) WWTP influent and effluent; and (**c**) hospital wastewater. Rows represent individual samples and columns represent ARGs. Relative gene abundance is indicated by a colour gradient from low (light green) to high (dark blue). Samples are grouped by sampling source, as indicated by the color bars.

ARGs associated with MLSB, tetracycline, and aminoglycoside resistance were detected across all wastewater sources (Figure 2). In community wastewater, the MLSB resistance gene *msrE* showed the highest relative gene abundance (mean ± SD: 8.65 × 10^−2^ ± 6.28 × 10^−2^). Other prevalent ARGs included the tetracycline resistance gene *tet(M)* (*n*/*N* = 72/72) and aminoglycoside resistance genes, including *aadA7*, *aadA2_3*, *AAC(6′)-Ib_1*, *aadA_1*, *APH(6)-Id*, and *ANT(2″)-Ia*. The methicillin resistance gene *mecA* was detected in 14 samples, with low relative gene abundance (mean ± SD: 6.39 × 10^−6^ ± 1.48 × 10^−5^). The quaternary ammonium resistance gene *qacH_1* was universally detected in all samples with high relative gene abundance (mean ± SD: 9.32 × 10^−3^ ± 6.16 × 10^−3^). ARG detection in WWTP influent broadly mirrored that observed in community wastewater, although the relative gene abundances were generally higher ranging from mean ± SD: 8.78 × 10^−2^ ± 4.45 × 10^−2^ to 2.90 × 10^−6^ ± 1.16 × 10^−5^. In WWTP effluent, ARGs representing several antibiotic classes, including aminoglycoside, MLSB, glycopeptide, and β-lactam resistance, remained detectable, although typically at lower relative gene abundances than in influent samples (Figure 2). Among the glycopeptide resistance genes, *vanA* was detected across all community, hospital, and WWTP samples and consistently showed higher relative gene abundance than *vanB*. The highest glycopeptide resistance gene abundances were observed in WWTP effluent, where *vanA* and *vanB* reached mean relative gene abundances of mean ± SD: 2.31 × 10^−2^ ± 1.94 × 10^−2^ and 2.31 × 10^−2^ ± 1.94 × 10^−2^, respectively. Compared with other wastewater sources, hospital wastewater showed higher ARG detection rates and greater relative gene abundance across multiple antibiotic classes. Aminoglycoside resistance genes were prominent, with *AAC(6′)-Ib_1* predominating with relative gene abundance (mean ± SD: 1.66 × 10^−1^ ± 1.26 × 10^−1^). For quinolone resistance, *qnrS2* was the most frequently detected marker (mean ± SD: 1.68 × 10^−2^ ± 1.50 × 10^−2^). Hospital wastewater also showed enrichment of β-lactam resistance genes, including *bla*_GES_, *bla*_TEM_, *bla*_KPC-2_, and *bla*_IMP-1_, which occurred at high relative gene abundance (mean ± SD: 4.46 × 10^−2^ ± 3.83 × 10^−2^ to 6.43 × 10^−3^ ± 4.66 × 10^−3^). In contrast to community wastewater, only one hospital wastewater sample showed a positive *mecA* signal (relative gene abundance: 2.93 × 10^−5^). Additional resistance markers detected across all wastewater sources included *qacEΔ1*, *merA*, and *bacA*, with the highest relative gene abundance observed in WWTP influent, followed by hospital wastewater (Supplementary Data S2). The colistin resistance gene *mcr-1* was also detected across all wastewater sources. Notably, *mcr-1* remained detectable in WWTP effluent and occurred at higher relative gene abundance (mean ± SD: 1.14 × 10^−3^ ± 1.85 × 10^−3^) compared to WWTP influent (2.58 × 10^−4^ ± 2.11 × 10^−4^) (Supplementary Data S2).

**Figure 3:**
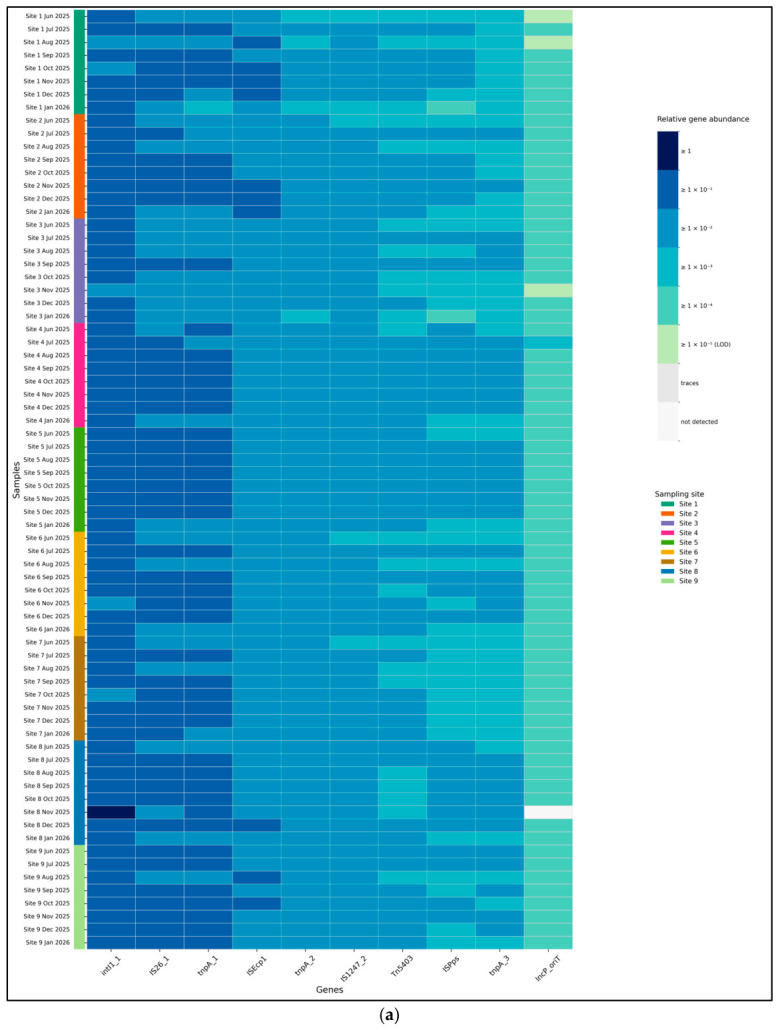

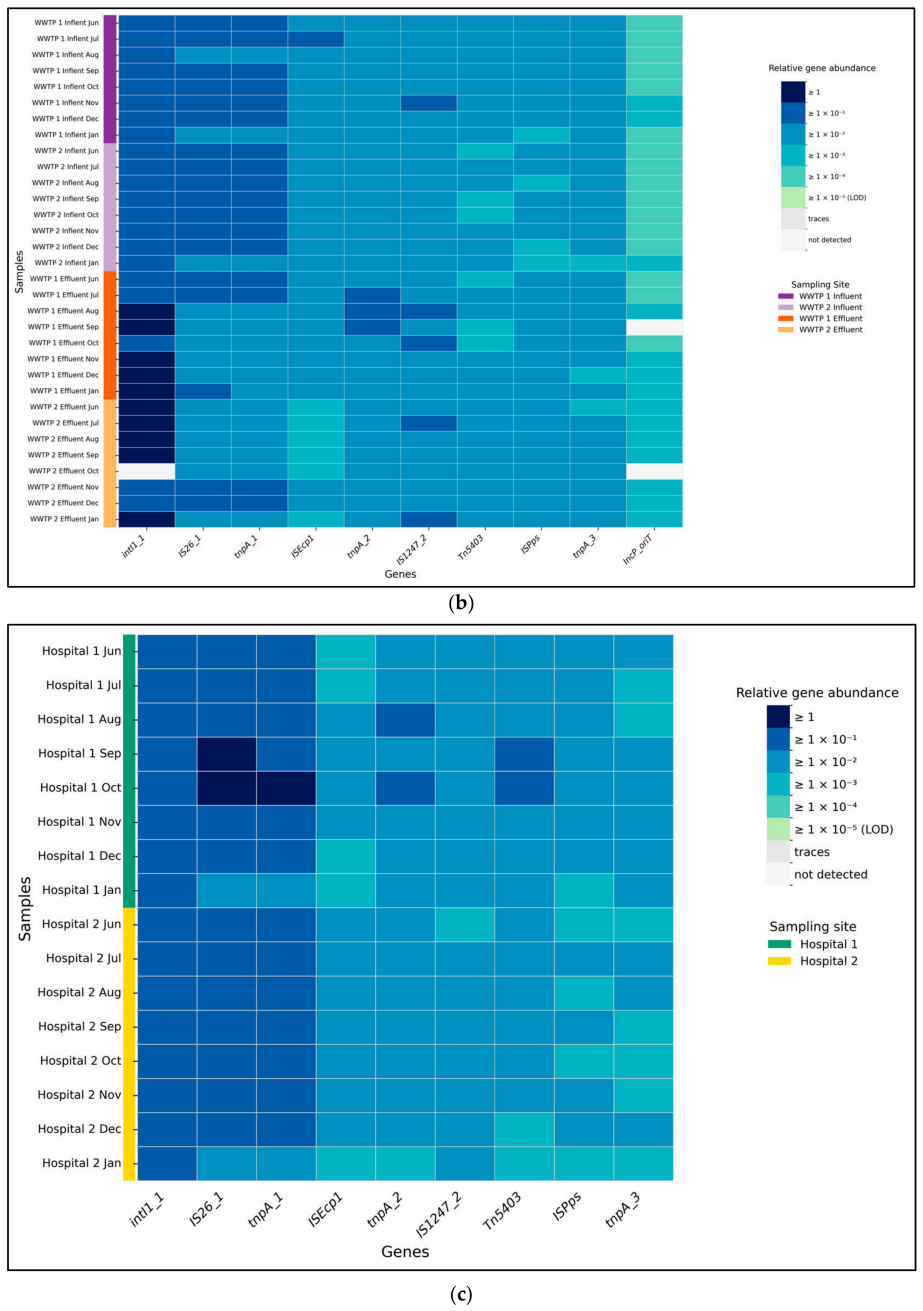
Distribution of relative abundance of mobile genetic elements across wastewater sources. Heatmap showing the relative abundances of mobile genetic elements (MGEs) markers based on HT-qPCR profiling, across wastewater sources: (**a**) community wastewater, (**b**) WWTP influent and effluent; and (**c**) hospital wastewater. Rows represent individual samples and columns represent MGEs. Relative gene abundance is indicated by a colour gradient from low (light green) to high (dark blue). Samples are grouped by sampling source, as indicated by the color bars.

MGEs were detected across all wastewater sources, although relative gene abundance differed across sources (Figure 3). WWTP effluent exhibited the highest overall MGE abundance, largely due to the strong dominance of *intI1_1* (mean ± SD: 2.93 ± 3.76), followed by WWTP influent (mean ± SD: 2.39 × 10^−1^ ± 1.33 × 10^−1^) (Figure 3). In hospital wastewater, *IS26_1* (mean ± SD: 4.05 × 10^−1^ ± 3.61 × 10^−1^) and *tnpA_1* (mean ± SD: 3.92 × 10^−1^ ± 3.10 × 10^−1^) were the most abundant MGEs, whereas community wastewater was dominated by *intI1_1* (mean ± SD: 1.95 × 10^−1^ ± 1.30 × 10^−1^), *IS26_1* (mean ± SD: 1.30 × 10^−1^ ± 7.074 × 10^−2^), and *tnpA_1* (mean ± SD: 1.13 × 10^−1^ ± 5.50 × 10^−2^). The other detected MGEs occurred at comparatively lower abundances across all sources, with *IncP_oriT* consistently showing the lowest relative gene abundance values.

### 2.3. Comparative Analysis and Network Correlation

**Figure 4:**
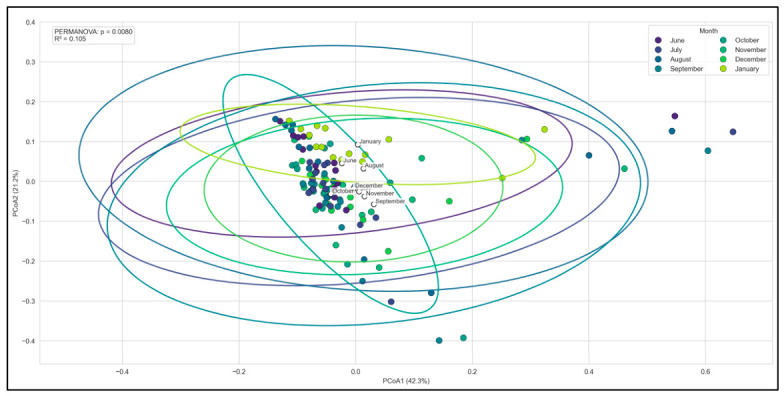
Temporal variation in wastewater relative abundance gene profiles. Principal coordinates analysis (PCoA) of Bray–Curtis dissimilarities based on relative abundance of HT-qPCR marker genes across 120 samples collected over an 8-month period. Each point represents an individual sample and is colored by sampling month. Ellipses indicate 95% confidence intervals for each month. Axes represent the first two principal coordinates (PCoA1 and PCoA2) with the percentage of variation explained shown in parentheses. Statistical significance of temporal variation was assessed using permutational multivariate analysis of variance (PERMANOVA) (R^2^ = 0.105, *p* = 0.008).

**Figure 5:**
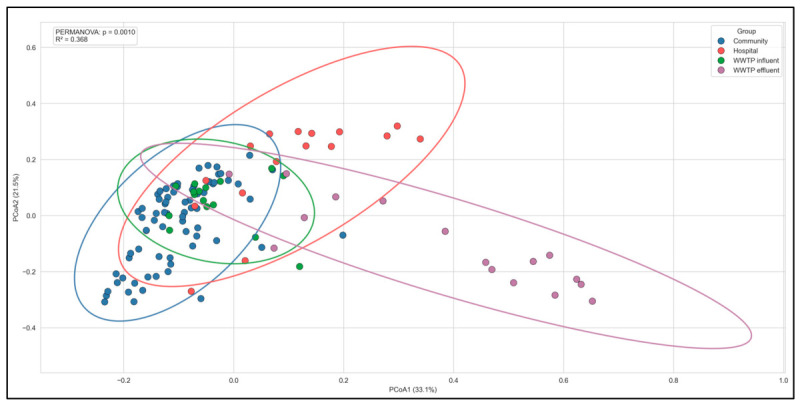
Variation in antibiotic resistance genes and mobile genetic elements across wastewater sources. Principal coordinates analysis (PCoA) of Bray–Curtis dissimilarities based on the relative abundance of antibiotic resistance genes (ARGs) and mobile genetic elements (MGEs) across wastewater sources (community, hospital, WWTP influent, and WWTP effluent). Each point represents an individual sample and is colored by sampling source. Ellipses indicate 95% confidence intervals for each group. Axes represent the first two principal coordinates (PCoA1 and PCoA2), with the percentage of variation explained shown in parentheses. Differences in gene composition between wastewater sources were assessed using permutational multivariate analysis of variance (PERMANOVA) (R^2^ = 0.368, *p* = 0.001).

PCoA based on Bray–Curtis dissimilarity showed that marker gene profiles across the 8-month sampling period largely overlapped, although PERMANOVA detected a statistically significant but modest effect of sampling month on overall gene composition (pseudo-F = 1.88, R^2^ = 0.105, *p* = 0.008) (Figure 4). In contrast, PCoA revealed clear differences in ARG and MGE profiles based on wastewater sources (Figure 5). Community wastewater formed a broad cluster that partially overlapped with WWTP influent, whereas hospital wastewater and WWTP effluent were more clearly separated along the main axes. PERMANOVA confirmed significant differences in gene composition among wastewater sources (pseudo-F = 22.54, R^2^ = 0.368, *p* = 0.001) (Figure 5).

**Figure 6:**
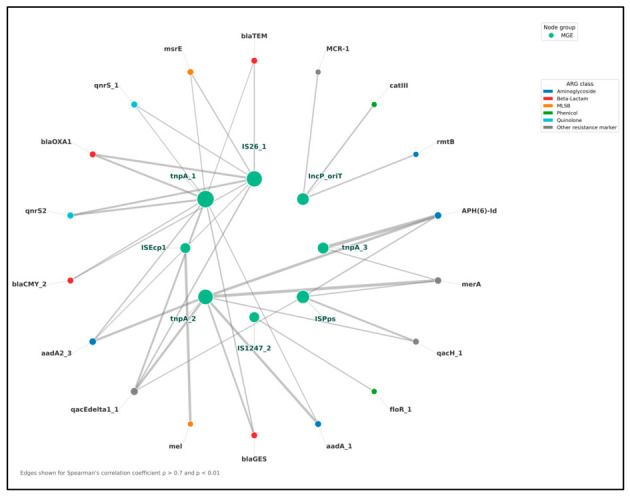
Co-occurrence network of antibiotic resistance genes and mobile genetic elements in wastewater. The network showing the significant co-occurrence relationships between antibiotic resistance genes (ARGs) and mobile genetic elements (MGEs) based on Spearman’s rank correlation analysis. The nodes represent ARGs and MGEs, with MGEs shown in green and ARGs coloured by antibiotic class. The edges represent statistically significant positive correlations (Spearman’s ρ > 0.7, *p* < 0.01), with edge thickness proportional to correlation strength. The node size is scaled according to degree (number of significant associations).

Correlation network analysis showed that a limited number of MGEs, including *tnpA_1*, *tnpA_2*, *tnpA_3*, *IS26_1*, *ISPps*, and *IncP_oriT*, acted as central hubs linking multiple ARGs (Figure 6). The strongest association was observed between *tnpA_3* and *APH(6)-Id* (ρ = 0.901). *tnpA_2* and *IS26_1* also showed multiple strong associations with *aadA*, *bla*_OXA-1_, *bla*_TEM_, *qnrS*, and *qacEΔ1*, while *IncP_oriT* was associated with *catIII*, *rmtB*, and *mcr-1* (Figure 6).

## 3. Discussion

Analysis of pathogen markers, ARGs, and MGEs across community, hospital, and WWTP compartments revealed distinct differences in composition and abundance. Hospital wastewater exhibited the highest diversity and relative abundance of resistance genes, whereas treated effluent showed reduced but persistent resistance signals. These patterns highlight wastewater systems as important resistome reservoirs and surveillance platforms. Although wastewater-based AMR surveillance has expanded globally, most studies originate from Europe, North America, and East Asia [20,21,22,23,24,25], leaving gaps in understanding resistance dynamics in globally connected regions such as the Arabian Gulf, where wastewater resistome data remain limited. This study provides baseline environmental AMR data and supports the integration of wastewater monitoring into broader AMR surveillance strategies.

The pathogen markers detected across wastewater compartments indicate strong human-associated microbial inputs into the sewer network. The consistent detection of enterococci, *E. coli*, and *K. pneumoniae* reflects fecal contributions from the urban population, while the greater detection frequency of *A. baumannii* and *P. aeruginosa* in hospital wastewater highlights the influence of clinical sources on wastewater microbial and resistance signatures. Across wastewater compartments, the resistome was dominated by ARGs associated with MLSB, aminoglycoside, and tetracycline antibiotic classes. The detection of glycopeptide resistance determinants, including *vanA* and *vanB*, was not unexpected given their established association with vancomycin-resistant *Enterococcus* spp. and the persistent detection of enterococcal markers across wastewater compartments.

This pattern is consistent with wastewater resistome studies from multiple regions, in which macrolide, aminoglycoside, and tetracycline resistance genes are among the most prevalent ARG classes in municipal wastewater systems [26]. These ARGs likely reflect long-term antimicrobial usage patterns in both clinical and community settings [27]. Healthcare environments are characterised by intensive antimicrobial usage and high densities of colonized or infected individuals, conditions that favour the selection and shedding of resistant microorganisms into wastewater systems [28]. Our findings demonstrated that hospital wastewater had the greatest diversity and relative abundance of ARGs, including enrichment of β-lactam resistance genes. This is consistent with previous studies reporting enrichment of clinically relevant resistance determinants in hospital effluents [13]. The detection of carbapenemase-associated β-lactam resistance genes in hospital wastewater highlights healthcare facilities as important point sources of clinically significant resistance genes entering municipal sewer networks.

Municipal sewer networks integrate microbial and genetic inputs from across urban environments. The similarity observed between community wastewater and WWTP influent profiles supports the concept that WWTP influent reflects aggregated microbial and genetic inputs from across the urban environment [7]. Wastewater treatment processes reduced the detection of pathogen markers and ARGs in WWTP effluent compared with WWTP influent although several resistance genes and MGEs remained detectable. Similar observations have been reported in wastewater studies where ARGs persist in WWTP effluent despite substantial reductions in microbial abundance [29]. Molecular detection of ARGs may represent DNA originating from viable bacteria, nonviable cells, or extracellular genetic material and therefore does not necessarily indicate active microbial populations [30]. Nevertheless, the persistence of ARGs and MGEs in WWTP effluent highlights the potential for wastewater discharge to contribute to environmental dissemination of resistance determinants through receiving water bodies and other environmental pathways. This is particularly relevant in regions where treated wastewater is increasingly reused for irrigation and other non-potable applications as part of water sustainability strategies [31]. Understanding the persistence and dynamics of resistance determinants in WWTP effluent is therefore important for balancing the benefits of utilizing treated wastewater with environmental and public health risk assessments.

Beyond antibiotic resistance determinants, the detection of genes associated with disinfectant and heavy metal resistance suggests that wastewater resistomes are influenced by multiple selective pressures. Genes such as *qacEΔ1*, commonly associated with integrons and resistance to quaternary ammonium compounds, may reflect co-selection driven by disinfectant exposure [32], while metal resistance genes indicate the potential influence of heavy metals and other environmental contaminants on ARG persistence [33]. Consistent with this, MGEs were widely detected across wastewater compartments, particularly in hospital wastewater and WWTP effluent.

The observed ARG–MGE co-occurrence patterns further support the role of horizontal gene transfer in shaping wastewater resistomes and facilitating ARG dissemination. Elements such as *IS26*-associated transposases and the class 1 integron integrase gene *intI1* have been widely implicated in the mobilization of multidrug resistance regions in Gram-negative bacteria [34,35]. These findings indicate that wastewater microbial communities represent dynamic environments in which antibiotic exposure and other anthropogenic chemical contaminants, including disinfectants, biocides, heavy metals, pharmaceutical residues, and industrial chemicals, may jointly create selective or co-selective pressures that influence the persistence and spread of antimicrobial resistance. While co-occurrence does not confirm physical linkage or direct horizontal gene transfer, these associations suggest shared ecological niches or potential mobility pathways that may contribute to the dissemination of resistance genes.

The PCoA results indicate that wastewater source was a major determinant of variation in marker gene composition, with hospital and WWTP effluent samples forming more distinct clusters than community samples and WWTP influent. In hospital wastewater, this pattern may be attributed to concentrated inputs associated with high antibiotic usage and patient-derived microbial loads [13]. The partial overlap between community samples and WWTP influent likely reflects the heterogeneous nature of influent wastewater, which integrates inputs from multiple sources including households, commercial activities, and healthcare facilities. The PERMANOVA result (R^2^ = 0.368) further supports the strong influence of sampling source on resistome structure. In contrast, temporal variation was weaker and the substantial overlap observed among monthly clusters, suggestive of a relatively stable resistome composition over time. However, sustained longitudinal work is needed to confirm this and clarify the impact of other factors, particularly seasonal influences such as temperature fluctuations.

HT-qPCR enabled rapid, scalable, and cost-effective detection of pathogen-associated markers, ARGs, and MGEs across wastewater compartments and is well-suited for large-scale surveillance of known resistance genes [16]. While HT-qPCR provides a targeted and sensitive overview of known resistance determinants, it cannot identify novel ARGs, resolve whether detected genes are located on chromosomes or mobile elements, or determine the bacterial hosts carrying these genes. Integration of shotgun metagenomics with chromosome conformation capture (Hi-C) sequencing would provide complementary insight into the taxonomic distribution, genomic organization, and mobility potential of resistance genes.

## 4. Materials and Methods

Study design: The study was conducted in Dubai, United Arab Emirates (UAE), with monthly sampling performed between June 2024 and January 2025. Wastewater samples were collected from three urban sources: community sewer nodes (nine pumping stations), tertiary-care hospital discharge points (two facilities sampled before sewer junctions), and two municipal wastewater treatment plants (WWTPs), each sampled at both influent and effluent stages. Sampling procedures followed the protocol previously described [36].

Sample preparation and DNA extraction: Wastewater (45 mL) was filtered using a Stericup Quick Release Vacuum Sterile Filtration System with a 0.22 µm polyethersulfone (PES) [36] membrane (Merck Millipore, Merck KGaA, Darmstadt, Germany). DNA was extracted using the DNeasy PowerWater Kit (QIAGEN, Hilden, Germany) according to the manufacturer’s instructions. Extracted DNA was quantified using a NanoDrop One spectrophotometer (Thermo Fisher Scientific, Waltham, MA, USA), and purity was assessed using A260/280 and A260/230 ratios prior to downstream molecular analyses.

HT-qPCR assay: Extracted DNA was screened using an HT-qPCR panel comprising 72 assays adapted from the Resistomap One Health AMR panel (Resistomap Oy, Helsinki, Finland), with modifications. The panel included 44 ARGs representing major antibiotic classes, including aminoglycosides, β-lactams, tetracyclines, macrolide–lincosamide–streptogramin B (MLSB), quinolones, sulfonamides, phenicols, and glycopeptides, ten MGE markers, and nine pathogen-associated markers alongside three taxonomic markers; and five additional resistance genes to provide environmental context. A single assay targeting the 16S rRNA gene was included in the panel as a universal bacterial marker and used as a reference target for normalization and relative quantification of ARG abundance. The list of targets and corresponding primer sequences is provided in Supplementary Data S1.

The HT-qPCR assays were performed using the SmartChip™ Real-Time PCR system (Takara Bio, San Jose, CA, USA) following the established Resistomap analytical workflow [37,38,39]. Briefly, 1728-well chips containing 100 nL reaction volumes were automatically loaded using the SmartChip Multisample Nanodispenser, with wastewater DNA samples and no-template controls included in parallel. Thermal cycling was performed at 95 °C for 10 min, followed by 40 cycles of 95 °C for 15 s and 60 °C for 60 s. Melt-curve analysis was conducted up to 97 °C with 0.4 °C increments.

Data processing and analysis: Amplification results were filtered based on melt-curve specificity and amplification quality, retaining detections only when Ct < 28 and amplification efficiency was within 2 ± 0.3 [40,41]. Technical replicates were evaluated for consistency, and replicates with a standard deviation greater than 0.5 or amplification efficiency deviating from 2 ± 0.1 were excluded. Targets detected in only one of three replicates were removed. The mean Ct of the remaining replicates was used to calculate ΔCt values relative to the 16S rRNA gene. Relative gene abundances were determined using the 2−ΔCt method (ΔCt = Ct target − Ct 16S rRNA) [42], and estimated copy numbers were calculated based on their abundance relative to the 16S rRNA gene and the corresponding 16S rRNA standard curve slope from the SmartChip run. Data processing and analysis were performed using the Resistomap analytical platform 2026.

Statistical analysis: Target-gene relative abundances were calculated by normalizing gene copy numbers to 16S rRNA gene abundance and summarized for each location across the 8-month sampling period as mean ± standard deviation (SD). To identify genes that differed significantly among sources, relative gene abundance (RA) values were log10-transformed [log_10_(RA + 10^−6^)] to reduce skewness. Differences among wastewater sources (community, hospital, WWTP influent, and WWTP effluent) were assessed using the Kruskal–Wallis test followed by Dunn’s post hoc pairwise comparisons with *p*-values adjusted using the Benjamini–Hochberg false discovery rate (FDR). Principal coordinates analysis (PCoA) was applied to the relative gene abundance matrix to evaluate differences in spatial and temporal compositional profiles across community wastewater, hospital, WWTP influent and WWTP effluent. Bray–Curtis dissimilarities were calculated from relative abundance data, and ordination was conducted using PCoA. Differences between groups were assessed using permutational multivariate analysis of variance (PERMANOVA) with 999 permutations. For visualization, samples were projected onto the first two principal coordinate axes, and 95% confidence ellipses were overlaid to illustrate group-level clustering. Associations between MGEs and ARGs were assessed using Spearman’s rank correlation. *p*-values were adjusted for multiple comparisons using the Benjamini–Hochberg false discovery rate, and significant positive correlations (ρ > 0.7, adjusted *p* < 0.01) were retained for network construction. The ARG–MGE network was visualized using a concentric layout, with MGEs at the center and ARGs arranged around them. Node size reflected the number of connections (degree), and edge width represented correlation strength.

## 5. Conclusions

In conclusion, this study provides insight into the structure and dynamics of the wastewater resistome across urban compartments. Using high-throughput qPCR and network analysis, we identify strong spatial differences, highlight hospital wastewater as a key source of clinically relevant resistance genes, and demonstrate the persistence of ARGs and MGEs in WWTP effluent. These findings support consideration of risk-based on-site pretreatment or source-control strategies at selected healthcare facilities, particularly where high antimicrobial use and specialized clinical activity may increase the discharge of resistance determinants. Such interventions should complement, rather than replace, effective municipal wastewater treatment and should be evaluated using monitoring frameworks that include viable microorganisms, ARGs, MGEs, pharmaceutical residues, and extracellular DNA. From a One Health perspective, wastewater networks act as convergence points where resistance determinants from different sources come together, providing insight into AMR circulation. High-throughput qPCR offers a practical and scalable approach for population-level AMR surveillance, with the potential to support early detection of emerging resistance and complement clinical surveillance systems. Integrating wastewater surveillance into national AMR frameworks, supported by expanded multi-omics and long-term sampling, will further strengthen its role as an early-warning platform.

## Data Availability

The original contributions presented in this study are included in the article/Supplementary Materials. Further inquiries can be directed to the corresponding author.

## Supplementary Materials

The following supporting information can be downloaded at https://www.mdpi.com/article/10.3390/antibiotics15070669/s1.

## Abbreviations

The following abbreviations are used in this manuscript:

AMR	Antimicrobial resistance
ARGs	Antibiotic resistance genes
HT-qPCR	High-throughput quantitative polymerase chain reaction
WWTP	Wastewater treatment plant
MGEs	Mobile genetic elements
MLSB	Macrolide–lincosamide–streptogramin B
qPCR	Quantitative polymerase chain reaction
PCR	Polymerase chain reaction
PCoA	Principal coordinates analysis
PERMANOVA	Permutational multivariate analysis of variance
SD	Standard deviation
RA	Relative abundance
FDR	False discovery rate
rRNA	Ribosomal RNA
Ct	Cycle threshold
UAE	United Arab Emirates
PES	Polyethersulfone
Hi-C	Chromosome conformation capture sequencing
NTC	No-template control
